# Defense against Adversarial Swarms with Parameter Uncertainty

**DOI:** 10.3390/s22134773

**Published:** 2022-06-24

**Authors:** Claire Walton, Isaac Kaminer, Qi Gong, Abram H. Clark, Theodoros Tsatsanifos

**Affiliations:** 1 Department of Electrical and Computer Engineering, University of Texas at San Antonio, San Antonio, TX 78249, USA; 2 Department of Mathematics, University of Texas at San Antonio, San Antonio, TX 78249, USA; 3 Department of Mechanical and Aerospace Engineering, Naval Postgraduate School, Monterey, CA 93943, USA; kaminer@nps.edu (I.K.); theodoros.tsatsanifos.gr@nps.edu (T.T.); 4 Department of Applied Mathematics, University of California Santa Cruz, Santa Cruz, CA 95064, USA; qigong@ucsc.edu; 5 Department of Physics, Naval Postgraduate School, Monterey, CA 93943, USA; abe.clark@nps.edu

**Keywords:** optimal control, parameter uncertainty, swarming

## Abstract

This paper addresses the problem of optimal defense of a high-value unit (HVU) against a large-scale swarm attack. We discuss multiple models for intra-swarm cooperation strategies and provide a framework for combining these cooperative models with HVU tracking and adversarial interaction forces. We show that the problem of defending against a swarm attack can be cast in the framework of uncertain parameter optimal control. We discuss numerical solution methods, then derive a consistency result for the dual problem of this framework, providing a tool for verifying computational results. We also show that the dual conditions can be computed numerically, providing further computational utility. Finally, we apply these numerical results to derive optimal defender strategies against a 100-agent swarm attack.

## 1. Introduction

Swarms are characterized by large numbers of agents which act individually, yet produce collective, herd-like behaviors. Implementing cooperating swarm strategies for a large-scale swarm is a technical challenge which can be considered to be from the “insider’s perspective”. It assumes inside control over the swarm’s operating algorithms. However, as large-scale ‘swarm’ systems of autonomous systems become achievable—such as those proposed by autonomous driving, UAV package delivery, and military applications—interactions with swarms outside our direct control become another challenge. This generates its own “outsider’s perspective” issues.

In this paper, we look at the specific challenge of protecting an asset against an adversarial swarm. Autonomous defensive agents are tasked with protected a high-value unit (HVU) from an incoming swarm attack. The defenders do not fully know the cooperating strategy employed by the adversarial swarm. Nevertheless, the task of the defenders is to maximize the probability of survival of the HVU against an attack by such a swarm. This challenge raises many issues—for instance, how to search for the swarm [1], how to observe and infer swarm operating algorithms [2], and how to best defend against the swarm given algorithm unknowns, and only limited, indirect control through external means. In this paper, we restrict ourselves to the last issue. However, these problems share multiple technical challenges. The preliminary approach we apply in this paper demonstrates some basic methods which we hope will stimulate the development of more sophisticated tools.

For objectives achieved via external control of the swarm, several features of swarm behavior must be characterized: capturing the dynamic nature of the swarm, tracking the collective risk profile created by a swarm, and engaging with a swarm via dynamic inputs, such as autonomous defenders. The many modeling layers create a challenge for generating an effective response to the swarm, as model uncertainty and model error are almost certain. In this paper, we look at several dynamic systems where the network structure is determined by parameters. These parameters set neighborhood relations and interaction rules. Additional parameters establish defender input and swarm risk.

We consider the generation of optimal defense strategies given uncertainty in parameter values. We demonstrate that small deviances in parameter values can have catastrophic effects on defense trajectories optimized without taking error into account. We then demonstrate the contrasting robustness of applying an uncertain parameter optimal control framework instead of optimizing with nominal values. The robustness against these parameter values suggests that refined parameter knowledge may not be necessary given appropriate computational tools. These computational tools—and the modeling of the high-dimensional swarm itself—are expensive. To assist with this issue, we provide dual conditions for this problem in the form of a Pontryagin minimum principle and prove the consistency of these conditions for the numerical algorithm. These dual conditions can, thus, be computed from the numerical solution of the computational method and provide a tool for solution verification and parameter sensitivity analysis.

Although in this paper, optimal strategies against swarms motivate the framework of uncertain parameter optimal control, and the subsequent development of the dual conditions, both the framework and the dual conditions have many applications beyond swarm defense. Optimal control with parameter uncertainty is relevant to robotics—where parts, such as wheels, may have small size and calibration uncertainties; aerospace—where both components and exogeneous factors, such as wind, may be modeled using parameter uncertainty; and search and rescue—where the location of a target object can be considered a parameter uncertainty [3,4]. It is also an instance of mean-field optimal control (which includes this framework, but also more general probability distributions), which is finding application in the training of neural networks [5]. The dual conditions provided in this paper provide both a tool for verification of numerical solutions, as well as another potential route for generating numerical solutions.

The structure of this paper is as follows. Section 2 provides examples of dynamic swarming models and extensions for defensive interactions. Section 3 discusses optimization challenges and describes a general uncertain parameter optimal control framework that this problem could be addressed with. Section 4 provides a proof of the consistency of the dual problem for this control framework, which expands on the results initially presented in the conference paper [6]. Section 5 gives an example of numerical implementation that demonstrates optimal defense against a large-scale swarm of 100 agents. Section 6 discusses the results and future work.

## 2. Modeling Adverserial Swarms

### 2.1. Cooperative Swarm Models

The literature on the design of swarm strategies which produce coherent, stable collective behavior has become vast. A quick review of the literature points to two main trends/categories in swarm behavior design. The first relies on dynamic modeling of the agents and potential functions to control their behavior (see [7,8] and references therein). The second trend relates to the use of rules to describe agents’ motion and local rule-based algorithms to control them [9,10].

We present two examples of dynamic swarming strategies from the literature. These examples are illustrative of the forces considered in many swarming models:

- collision avoidance between swarm members;
- alignment forces between neighboring swarm members;
- stabilizing forces.

These intra-swarm goals are aggregated to provide a swarm control law, which we will refer to as $F_{S}$, to each swarm agent. Both example models in this paper share the same double integrator form with respect to this control law. For *n* swarm agents, the dynamics are defined by

(1) $$\begin{array}{cc} \; & {\ddot{x}}_{i}=u_{i}.\;\;i=1,\ldots ,n, \end{array}$$

(2) $$u_{i}=F_{S}\left(x_{i},{\dot{x}}_{i},\forall j\neq i:x_{j},{\dot{x}}_{j}|\theta \right).$$

#### 2.1.1. Example Model 1: Virtual Body Artificial Potential

In this model [11,12], swarm agents track to a virtual body (or bodies) guiding their course, while also reacting to intra-swarm forces of collision avoidance and group cohesion. The input $u_{i}$ is the sum of intra-swarm forces, virtual body tracking, and a velocity dampening term. In addition, in this adversarial scenario, swarm agents are influenced to avoid intruding defense agents. The intra-swarm force between two swarm agents has magnitude $f_{I}$ and is a gradient of an artificial potential $V_{I}$. Let

(3) $$x_{ij}=x_{i}-x_{j}.$$

The artificial potential $V_{I}$ depends on the distance $\left|\right|x_{ij}\left|\right|$ between swarm agents *i* and *j*. The artificial potential $V_{I}$ is defined as:

(4) $$V_{I}=\left\{\begin{array}{c} \alpha \left(\ln\left(\left|\right|x_{ij}\left|\right|\right)+\frac{d_{0}}{\left|\right|x_{ij}\left|\right|}\right),\;\;\;0<||x_{ij}\left|\right|<d_{1}\\ \alpha \left(\ln\left(d_{1}\right)+\frac{d_{0}}{d_{1}}\right),\;\;\;\;\;\;\;\;\;\;\;\;\;||x_{ij}\left|\right|\geq d_{1} \end{array}\right.$$

where α is a scalar control gain, and $d_{0}$ and $d_{1}$ are scalar constants for distance ranges. Then the magnitude of interaction force is given by

(5) $$f_{I}=\left\{\begin{array}{c} \nabla_{\left|\right|x_{ij}\left|\right|}V_{I},\;\;\;0<||x_{ij}\left|\right|<d_{1}\\ 0,\;\;\;\;\;\;\;\;\;\;\;\;\;||x_{ij}\left|\right|\geq d_{1} \end{array}\right.$$

The swarm body is guided by ‘virtual leaders’, non-corporeal reference trajectories which lead the swarm. We assign a potential $V_{h}$ on a given swarm agent *i* associated with the *k*-th virtual leader, defined with the distance $\left|\right|h_{ik}\left|\right|$ between the swarm agent *i* and leader *k*. Mirroring the parameters α, $d_{0}$, and $d_{1}$ defining $V_{I}$, we assign $V_{h}$ the parameters $\alpha_{h}$, $h_{0}$, and $h_{1}$. An additional dissipative force $f_{v_{i}}$ is included for stability. The control law $u_{i}$ for the vehicle *i* associated with *m* defenders is given by

(6) $$\begin{array}{cc} u_{i} & =-\sum_{j\neq i}^{n}\nabla_{x_{i}}V_{I}\left(x_{ij}\right)-\sum_{k=1}^{m}\nabla_{x_{i}}V_{h}\left(h_{ik}\right)+f_{v_{i}}\\ \; & =-\sum_{j\neq i}^{n}\frac{f_{I}\left(x_{ij}\right)}{\left|\right|x_{ij}\left|\right|}x_{ij}-\sum_{k=1}^{m}\frac{f_{h}\left(h_{ik}\right)}{\left|\right|h_{ik}\left|\right|}h_{ik}+f_{v_{i}}. \end{array}$$

#### 2.1.2. Example Model 2: Reynolds Boid Model

In this model [8,13], for radius *r*, j=1,⋯,N, define the neighbors of agent *i* at position $x_{i}\in R^{n}$ by the set

(7) $$N_{i}=\{j|j\neq i\wedge ∥x_{i}-x_{j}∥<r\}$$

Swarm control is designated by three forces.

Alignment of velocity vectors:

(8) $$f_{al}=-w_{al}\left({\dot{x}}_{i}-\frac{1}{|N_{i}|}\sum_{j\in N_{i}}{\dot{x}}_{j}\right)$$

Cohesion of swarm:

(9) $$f_{coh}=-w_{coh}\left(x_{i}-\frac{1}{|N_{i}|}\sum_{j\in N_{i}}x_{j}\right)$$

Separation between agents:

(10) $$f_{sep}=-w_{sep}\frac{1}{|N_{i}|}\left(\sum_{j\in N_{i}}\frac{x_{j}-x_{i}}{∥x_{i}-x_{j}∥}\right)$$

for positive constant parameters $w_{al}$, $w_{coh}$, $w_{sep}$.

(11) $$u_{i}=f_{al}+f_{coh}+f_{sep}$$

### 2.2. Adversarial Swarm Models

The previous subsection provides several examples of inner swarm cooperative forces, $F_{S}$. In order to enable adversarial behavior and defense, these inner swarm cooperative forces need to be supplemented by additional forces of exogenous input into the collective. As written, the above cooperative swarming models neither respond to outside agents nor ‘attack’ (swarm towards) a specific target. We, thus, supplement the control laws above with two additional forces. The first, we refer to as $F_{HVU}$; the goal of the swarm, in this paper, is limited to tracking an HVU. An example of $F_{HVU}$ is provided in the example of Section 5, in Equation (28).

We also supplement by an adversarial force, which we refer to as $F_{D}$. The review [7] discusses several approaches to adversarial control. Examples include containment strategies modeled after dolphins [14], sheep-dogs [15,16], and birds of prey [17]. In [18], the authors studied the interaction between two swarms, one of which could be considered adversarial. In these examples of adversarial swarm control, the mechanism of interaction and defense is provided through the swarm’s own pursuit and evasion responses. This indirectly uses the swarm’s own response strategy against it—an approach which can be termed ‘herding’.

In addition to herding reactions, one can consider more direct additional forces of disruption, to model neutralizing swarm agents and/or physically remove them from the swarm. One form this can take, for example, is the removal of agents from the communications network, as considered in [19]. Another approach is taken in [20], which uses survival probabilities based on damage attrition. Defenders and the attacking swarm engage in mutual damage attrition while the swarm also damages the HVU when in proximity to it. Probable damage between agents is tracked as damage rates over time, where the rate of damage is based on features such as distance between agents and angle of attack. The damage rate at time *t* provides the probability of a successful ‘hit’ in time period [t,t+Δt]. The probability of agent survival can be modeled based on the aggregate number of hits it takes to incapacitate the agent. The authors of [20] provide derivations for multiple possibilities, such as single-shot destruction and N-shot destruction. These probabilities take the form of ODE equations. Tracking survival probabilities thus adds an additional state to the dynamics of each agent—a survival probability state.

We, thus, summarize a control scheme with HVU target-tracking and herding driven by the reactive forces of collision avoidance with the defenders as the following, for HVU states $y_{0}$ and defender states $y_{k}$, k=1,⋯,K:

(12) $$\begin{array}{ccc} u_{i}= & F_{S}\left(x_{i},{\dot{x}}_{i},\forall j\neq i:x_{j},{\dot{x}}_{j}|\theta \right) & \leftarrow \mathrm{intra-swarm}\\ + & F_{HVU}\left(x_{i},{\dot{x}}_{i},y_{0},{\dot{y}}_{0}|\theta \right) & \leftarrow \mathrm{target tracking}\\ + & F_{D}\left(x_{i},{\dot{x}}_{i},\forall k:y_{k},{\dot{y}}_{k}|\theta \right) & \leftarrow \mathrm{herding and/or damage} \end{array}$$

#### Example Attrition Model: Single-Shot Destruction

From [20]: let $P_{0}\left(t\right)$ be the probability the HVU has survived up to time *t*, $P_{k}\left(t\right)$, k=1,⋯,K, the probability defender *k* has survived, and $Q_{j}\left(t\right)$, j=1,⋯,N the probability swarm attacker *j* has survived. Let $d_{y}^{j,k}\left(x_{j}\left(t\right),y_{k}\left(t\right)\right)$ be the damage the defender $y_{k}$ inflicts on swarm attacker $x_{j}$ and let $d_{x}^{k,j}\left(y_{k}\left(t\right),x_{j}\right)$ be the damage the swarm attacker $x_{j}$ inflicts on the defender $y_{k}$, with the HVU represented by k=0.

Then the survival probabilities for attackers and defenders from single-shot destruction are given by the coupled ODEs:

$$\left\{\begin{array}{cc} {\dot{Q}}_{j}\left(t\right)=-Q_{j}\left(t\right)\sum_{k=1}^{K}P_{k}\left(t\right)d_{y}^{j,k}\left(x_{j}\left(t\right),y_{k}\left(t\right)\right), & Q_{j}\left(0\right)=1\\ {\dot{P}}_{k}\left(t\right)=-P_{k}\left(t\right)\sum_{j=1}^{N}Q_{j}\left(t\right)d_{x}^{k,j}\left(y_{k}\left(t\right),x_{j}\left(t\right)\right), & P_{k}\left(0\right)=1 \end{array}\right.$$

for j=1,⋯,N, k=0⋯,K.

## 3. Problem Formulation

**Figure 1:**
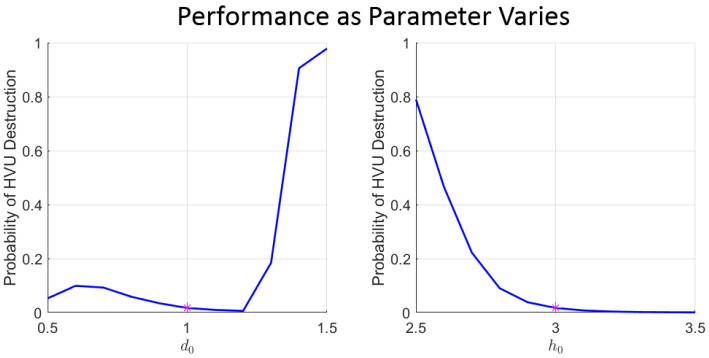
Example performance of solutions calculated using nominal values when parameter value is varied. Calculated using values in Section 5.1. Magenta dot marks the nominal value used in the optimization problem. In the left panel, $d_{0}$ is varied as the parameter; in the right panel $h_{0}$ is varied.

The above models depend on a large number of parameters. The dynamic swarming model coupled with attrition functions results in over a dozen key parameters, and many more would result from a non-homogeneous swarm. A concern would be that this adds too much model specificity, making optimal defense strategies lack robustness due to sensitivity to the specific set of model parameters. This concern turns out to be justified. When defense strategies are optimized for fixed, nominal parameter values, they display catastrophic failure for small perturbations of certain parameters, as can be seen in Figure 1. In fact, the plots included in Figure 1 clearly demonstrate that the sensitivity of the cost with respect to the uncertain parameters is highly non-linear. Thus, generating robust defense strategies requires a more sophisticated formalism introduced in the next Section 3.1.

### 3.1. Uncertain Parameter Optimal Control

The class of problems addressed by the computational algorithm is defined as follows:

**Problem P.**  Determine the function pair (x,u) with $x\in W_{1,\infty }\left(\left[0,T\right]\times \Theta ;R^{n_{x}}\right)$, $u\in L_{\infty }\left(\left[0,T\right];R^{n_{u}}\right)$ that minimizes the cost

(13) $$\begin{array}{c} J\left[x,u\right]=\;\;\int_{\Theta }\;\left[F\left(x(T,\theta ),\theta \right)\;+\;\;\int_{0}^{T}\;r\left(x\left(t,\theta \right),u\left(t\right),t,\theta \right)dt\right]d\theta \end{array}$$

subject to the dynamics

(14) $$\begin{array}{cc} \frac{\partial x}{\partial t}\left(t,\theta \right) & =f(x(t,\theta ),u(t),\theta ), \end{array}$$

initial condition $x\left(0,\theta \right)=x_{0}\left(\theta \right)$, and the control constraint g(u(t))≤0 for all t∈[0,T]. The set $L_{\infty }\left(\left[0,T\right];R^{n_{u}}\right)$ is the set of all essentially bounded functions, $W_{1,\infty }\left(\left[0,T\right]\times \Theta ;R^{n_{x}}\right)$ the Sobolev space of all essentially bounded functions with essentially bounded distributional derivatives, and $F:R^{n_{x}}\times R^{n_{\theta }}\mapsto R$, $r:R^{n_{x}}\times R^{n_{u}}\times R\times R^{n_{\theta }}\mapsto R$, $g:R^{n_{u}}\mapsto R^{n_{g}}$. Additional conditions imposed on the state and control space and component functions are specified in Appendix A.

In Problem P, the set Θ is the domain of a parameter $\theta \in R^{n_{\theta }}$. The format of the cost functional is that of the integral over Θ of a Mayer–Bolza type cost with parameter θ. This parameter can represent a range of values for a feature of the system, such as in ensemble control [21], or a stochastic parameter with a known probability density function.

For computation of numerical solutions, we introduce an approximation of Problem P, referred to as Problem $P^{M}$. Problem $P^{M}$ is created by approximating the parameter space, Θ, with a numerical integration scheme. This numerical integration scheme is defined in terms of a finite set of *M* nodes ${\left\{\theta_{i}^{M}\right\}}_{i=1}^{M}$ and an associated set of *M* weights ${\left\{\alpha_{i}^{M}\right\}}_{i=1}^{M}\subset R$ such that

(15) $$\int_{\Theta }h\left(\theta \right)d\theta =\lim_{M\to \infty }\sum_{i=1}^{M}h\left(\theta_{i}^{M}\right)\alpha_{i}^{M}.$$

given certain function smoothness assumptions. See Appendix A Assumption A1 for formal assumptions. Throughout the paper, *M* is used to denote the number of nodes used in this approximation of parameter space.

For a given set of nodes ${\left\{\theta_{i}^{M}\right\}}_{i=1}^{M}$, and control u(t), let ${\bar{x}}_{i}^{M}\left(t\right)$, i=1,⋯,M, be defined as the solution to the ODE created by the state dynamics of Problem P evaluated at $\theta_{i}^{M}$:

(16) $$\begin{array}{c} \left\{\begin{array}{c} \frac{d{\bar{x}}_{i}^{M}}{dt}\left(t\right)=f\left({\bar{x}}_{i}^{M}\left(t\right),u\left(t\right),\theta_{i}^{M}\right)\\ {\bar{x}}_{i}^{M}\left(0\right)=x_{0}\left(\theta_{i}^{M}\right), \end{array}\right.\;i=1,\cdots ,M. \end{array}$$

Let ${\bar{X}}^{M}\left(t\right)=\left[{\bar{x}}_{1}^{M}\left(t\right),\ldots ,{\bar{x}}_{M}^{M}\left(t\right)\right]$. The system of ODEs defining ${\bar{X}}^{M}$ has dimension $n_{x}\times M$, where $n_{x}$ is the dimension of the original state space and *M* is the number of nodes. The numerical integration scheme for parameter space creates an approximate objective functional, defined by:

(17) $${\bar{J}}^{M}\left[{\bar{X}}^{M},u\right]\;=\;\sum_{i=1}^{M}\left[F\left({\bar{x}}_{i}^{M}\left(T\right),\theta_{i}^{M}\right)+\int_{0}^{T}r\left({\bar{x}}_{i}^{M}\left(t\right),u\left(t\right),t,\theta_{i}^{M}\right)dt\;\right]\alpha_{i}^{M}.$$

In [4], the consistency of $P^{M}$ is proved. This is the property that, if optimal solutions to Problem $P^{M}$ converge as the number of nodes M→∞, they converge to feasible, optimal solutions of Problem P. See [4] for detailed proof and assumptions.

### 3.2. Computational Efficiency

The computation time of the numerical solution to the discretized problem defined in Equations (16) and (17) will depend on the value of *M*. Ideally, it should be sufficiently small so as to allow for a fast solution. On the other hand, a value of *M* that is too small will result in a solution that is not particularly useful, i.e., too far from the optimal. Naturally, the question arises: how far is a particular solution from the optimal? One tool for assessing this lies in computing the Hamiltonian and is addressed in Section 4.

## 4. Consistency of Dual Variables

The dual variables provide a method to determine the solution of an optimal control problem or a tool to validate a numerically computed solution. For numerical schemes based on direct discretization of the control problem, analyzing the properties of the dual variables and their resultant Hamiltonian may also lead to insight into the validity of an approximation scheme [22,23]. This could be especially helpful in high-dimensional problems, such as swarming, where parsimonious discretization is crucial to computational tractability.

**Figure 2:**
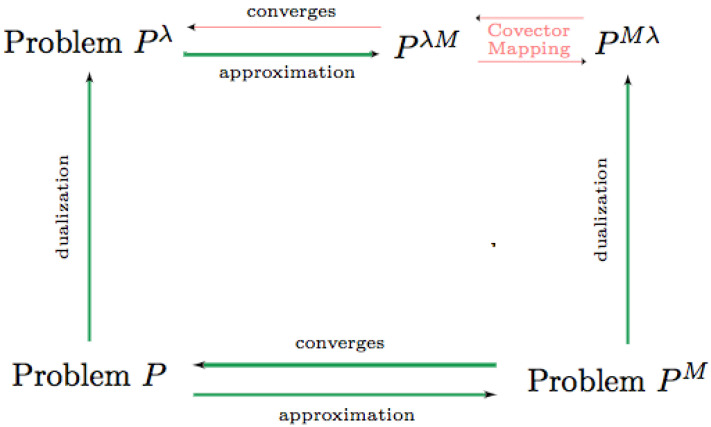
Diagram of primal and dual relations for parameter uncertainty control. Red lines designate the contribution of this paper.

Previous work shows the consistency of the primal variables in approximate Problem $P^{M}$ to the original parameter uncertainty framework of Problem P. Here, we build on that and prove the consistency of the dual problem of Problem P as well. This theoretical contribution is diagrammed in Figure 2. The consistency of the dual problem in parameter space enables approximate computation of the Hamiltonian from numerical solutions.

In [24], necessary conditions for Problem P were established. These conditions are as follows:

**Problem**$P^{\lambda }$  [([24], pp. 80–82)]. If $\left(x^{*},u^{*}\right)$ is an optimal solution to Problem P, then there exists an absolutely continuous costate vector $\lambda^{*}\left(t,\theta \right)$, such that for θ∈Θ:

$$\frac{\partial \lambda^{*}}{\partial t}\left(t,\theta \right)=-\frac{\partial H(x^{*},\lambda^{*},u^{*},t,\theta )}{\partial x},$$

(18) $$\lambda^{*}\left(T,\theta \right)=\frac{\partial F(x^{*}\left(T,\theta \right),\theta )}{\partial x}$$

where *H* is defined as:

H(x,λ,u,t,θ)=

(19) λf(x(t,θ),u(t),θ)+r(x(t,θ),u(t),t,θ).

Furthermore, the optimal control $u^{*}$ satisfies

$$u^{*}\left(t\right)=\underset{u\in U}{\arg\min}H\left(x^{*},\lambda^{*},u,t\right),$$

where H is given by

(20) $$H\left(x,\lambda ,u,t\right)=\int_{\Theta }H\left(x,\lambda ,u,t,\theta \right)d\theta .$$

Because Problem $P^{M}$ is a standard non-linear optimal control problem, it admits a dual problem as well. Problem $P^{M\lambda }$, provided by the Pontryagin minimum principle (a survey of minimum principle conditions is given by [25]). Applied to $P^{M}$ this generates:

**Problem $P^{M\lambda }$.**  For feasible solution $\left({\bar{X}}^{M},u\right)$ to Problem $P^{M}$, find $\bar{\Lambda }\left(t\right)=\left[{\bar{\lambda }}_{1}^{M}\left(t\right),\cdots {\bar{\lambda }}_{M}^{M}\left(t\right)\right]$, ${\bar{\lambda }}_{i}^{M}:\left[0,T\right]\to R^{N_{x}}$, that satisfies the following conditions:

$$\frac{d{\bar{\lambda }}_{i}^{M}}{dt}\left(t\right)=-\frac{\partial {\bar{H}}^{M}\left({\bar{x}}_{i}^{M},{\bar{\lambda }}_{i}^{M},u,t\right)}{\partial x_{i}^{M}},$$

(21) $${\bar{\lambda }}_{i}^{M}\left(T\right)=\alpha_{i}^{M}\frac{\partial F({\bar{x}}_{i}^{M},\theta_{i}^{M})}{\partial {\bar{x}}_{i}^{M}},$$

where ${\bar{H}}^{M}$ is defined as:

(22) $${\bar{H}}^{M}\left({\bar{X}}^{M},{\bar{\Lambda }}_{M},u,t\right)=\sum_{i=1}^{M}\left[{\bar{\lambda }}_{i}^{M}f\left({\bar{x}}_{i}^{M}\left(t\right),u\left(t\right),\theta_{i}^{M}\right)+\alpha_{i}^{M}r\left({\bar{x}}_{i}^{M}\left(t\right),u\left(t\right),t,\theta_{i}^{M}\right)\right].$$

An alternate direction from which to approach solving Problem P overall is to approximate the necessary conditions of Problem P, i.e., Problem $P^{\lambda }$, directly rather than to approximate Problem P. This creates the system of equations:

$$\frac{d\lambda }{dt}\left(t,\theta_{i}^{M}\right)=-\frac{\partial H(x,u,t,\theta_{i}^{M})}{\partial x}$$

(23) $$\lambda \left(T,\theta_{i}^{M}\right)=\frac{\partial F(x\left(T,\theta_{i}^{M}\right),\theta_{i}^{M})}{\partial x}$$

for i=1,⋯,M, where *H* is defined as:

H(x,λ,u,t,θ)=λf(x(t,θ),u(t),θ)+r(x(t,θ),u(t),t,θ).

This system of equations can be re-written in terms of the quadrature approximation of the stationary Hamiltonian defined in Equation (20). Define

$${\tilde{H}}^{M}\left(x,\lambda ,u,t\right):=\sum_{i=1}^{M}\alpha_{i}^{M}H\left(x\left(t,\theta_{i}^{M}\right),\lambda \left(t,\theta_{i}^{M}\right),u\left(t\right),t,\theta_{i}^{M}\right).$$

Let

$$\tilde{\Lambda }\left(t\right)=\left[{\tilde{\lambda }}_{1}^{M}\left(t\right),\cdots {\tilde{\lambda }}_{M}^{M}\left(t\right)\right]=\left[\lambda \left(t,\theta_{1}^{M}\right),\cdots ,\lambda \left(t,\theta_{M}^{M}\right)\right]$$

and let

$${\tilde{X}}_{M}=\left[{\tilde{x}}_{1}^{M}\left(t\right),\cdots ,{\tilde{x}}_{M}^{M}\left(t\right)\right]$$

denote the semi-discretized states from Equation (16). Equation (23) can then be written as:

$$\frac{d{\tilde{\lambda }}_{i}^{M}}{dt}\left(t\right)=-\frac{1}{\alpha_{i}^{M}}\cdot \frac{\partial {\tilde{H}}^{M}\left({\tilde{X}}_{M},\tilde{\Lambda },u,t\right)}{\partial {\tilde{x}}_{i}^{M}}$$

(24) $${\tilde{\lambda }}_{i}^{M}\left(T\right)=\frac{\partial F({\tilde{x}}_{i}^{M}\left(T\right),\theta_{i}^{M})}{\partial {\tilde{x}}_{i}^{M}}$$

for i=1,⋯,M. Thus, we reach the following discretized dual problem:

**Problem $P^{\lambda M}$.**  For feasible controls *u* and solutions ${\tilde{X}}_{M}$ to Equation (16), find $\tilde{\Lambda }\left(t\right)=\left[{\tilde{\lambda }}_{1}^{M}\left(t\right),\cdots {\tilde{\lambda }}_{M}^{M}\left(t\right)\right]$, ${\tilde{\lambda }}_{i}^{M}:\left[0,T\right]\to R^{n_{x}}$, that satisfies the following conditions:

$$\frac{d{\tilde{\lambda }}_{i}^{M}}{dt}\left(t\right)=-\frac{1}{\alpha_{i}^{M}}\cdot \frac{\partial {\tilde{H}}^{M}\left({\tilde{X}}_{M},\tilde{\Lambda },u,t\right)}{\partial {\tilde{x}}_{i}^{M}},$$

(25) $${\tilde{\lambda }}_{i}^{M}\left(T\right)=\frac{\partial F({\tilde{x}}_{i}^{M},\theta_{i}^{M})}{\partial {\tilde{x}}_{i}^{M}},$$

where ${\tilde{H}}^{M}$ is defined as:

(26) $${\tilde{H}}^{M}\left({\tilde{X}}_{M},{\tilde{\Lambda }}_{M},u,t\right)=\sum_{i=1}^{M}\left[\alpha_{i}^{M}{\tilde{\lambda }}_{i}^{M}f\left({\tilde{x}}_{i}^{M}\left(t\right),u\left(t\right),\theta_{i}^{M}\right)+\alpha_{i}^{M}r\left({\tilde{x}}_{i}^{M}\left(t\right),u\left(t\right),t,\theta_{i}^{M}\right)\right].$$

**Lemma** **1.** *The mapping:*

$$\left({\bar{x}}_{i}^{M},\bar{u}\right)\mapsto \left({\tilde{x}}_{i}^{M},\tilde{u}\right),\;\frac{{\bar{\lambda }}_{i}^{M}}{\alpha_{i}^{M}}\mapsto {\tilde{\lambda }}_{i}^{M},$$

*for i=1,⋯,M is a bijective mapping from solutions of Problem $P^{M\lambda }$ to Problem $P^{\lambda M}$.*

**Proof.**  In the case of this particular problem, unlike standard control, the collocation of the relevant dynamics involves no approximation of differentiation (since the discretization is in the parameter domain rather than the time domain), and, thus, the mapping of covectors between Problem $P^{M\lambda }$ and Problem $P^{{\tilde{H}}^{M}\left({\tilde{X}}_{M},{\tilde{\Lambda }}_{M},u,t\right)=\lambda M}$ is straightforward and simply constructively provided by the lemma itself. The two mappings of the lemma, $\left({\bar{x}}_{i}^{M},\bar{u}\right)\mapsto \left({\tilde{x}}_{i}^{M},\tilde{u}\right)$ (identity mapping) and $\frac{{\bar{\lambda }}_{i}^{M}}{\alpha_{i}^{M}}\mapsto {\tilde{\lambda }}_{i}^{M}$ (scaling by $\frac{1}{\alpha_{i}^{M}}$) are both bijections. □

**Theorem** **1.** *Let ${\left\{{\tilde{X}}_{M},{\tilde{\Lambda }}_{M},u_{M}\right\}}_{M\in V}$ be a sequence of solutions for Problem $P^{\lambda M}$ with an accumulation point $\left\{{\tilde{X}}^{\infty },{\tilde{\Lambda }}^{\infty },u^{\infty }\right\}$. Let $\left(x^{\infty },\lambda^{\infty },u^{\infty }\right)$ be the solutions to Problem $P^{\lambda }$ for the control $u^{\infty }$. Then*

$$\lim_{M\in V}{\tilde{H}}^{M}\left({\tilde{X}}_{M},{\tilde{\Lambda }}_{M},u_{M},t\right)=H\left(x^{\infty },\lambda^{\infty },u^{\infty },t\right)$$

*where ${\tilde{H}}^{M}$ is the Hamiltonian of Problem $P^{\lambda M}$ as defined by Equation (26) and H is the Hamiltonian of Problem P as defined by Equation (20). The proof of this theorem can be found in the Appendix B.*

The convergence of the Hamiltonians of the approximate, standard control problems to the Hamiltonian of the general problem, $H(x^{\infty },\lambda^{\infty },u^{\infty },t)$, means that many of the useful features of the Hamiltonians of standard optimal control problems are preserved. For instance, it is straightforward to show that the satisfaction of Pontryagin’s minimum principle by the approximate Hamiltonians implies minimization of $H(x^{\infty },\lambda^{\infty },u^{\infty },t)$ as well. That is, that

$$H\left(x^{\infty },\lambda^{\infty },u^{\infty },t\right)\leq H\left(x^{\infty },\lambda^{\infty },u,t\right)$$

for all feasible *u*. Furthermore, when applicable, the stationarity properties of the standard control Hamiltonian, such as a constant-valued Hamiltonian in time-invariant problems, or stationarity with respect to u(t) in problems with open control regions, are also preserved.

## 5. Numerical Example

In a slight refashioning of the notation in the Section 2.2, Equation (12), let the parameter vector θ be defined by all the *unknown* parameters defining the interaction functions. Assuming prior distribution ϕ(θ) over these unknowns and parameter bounds Θ, we construct the following optimal control problem for robustness against the unknown parameters.

**Problem SD** (Swarm Defense)**.**  For *K* defenders and *N* attackers, determine the defender controls $u_{k}\left(t\right)$ that minimize:

(27) $$J=\int_{\theta }\left[1-P_{0}\left(t_{f},\theta \right)\right]\phi \left(\theta \right)d\theta$$

subject to:

$$\left\{\begin{array}{cc} {\dot{y}}_{k}\left(t\right)=f\left(y_{k}\left(t\right),u_{k}\left(t\right)\right), & \;y_{k}\left(0\right)=y_{k0}\\ {\ddot{x}}_{j}\left(t,\theta \right)= & \\ F_{S}\left(t,\theta \right)+F_{HVU}\left(t,\theta \right)+F_{D}\left(t,\theta \right), & \;x_{j}\left(0,\theta \right)=x_{j0}\left(\theta \right)\\ {\dot{Q}}_{j}\left(t,\theta \right)= & \\ -Q_{j}\left(t,\theta \right)\sum_{k=1}^{K}P_{k}\left(t,\theta \right)d_{y}^{j,k}\left(x_{j}\left(t,\theta \right),y_{k}\left(t\right)\right), & \;Q_{j}\left(0,\theta \right)=1\\ {\dot{P}}_{k}\left(t\right)= & \\ -P_{k}\left(t,\theta \right)\sum_{j=1}^{N}Q_{j}\left(t,\theta \right)d_{x}^{k,j}\left(y_{k}\left(t\right),x_{j}\left(t,\theta \right)\right), & \;P_{k}\left(0,\theta \right)=1 \end{array}\right.$$

for swarm attackers j=1,⋯,N and controlled defenders k=1⋯,K.

We implement Problem **SD** for both swarm models in Section 2.1, for a swarm of N=100 attackers and K=10 defenders.

### 5.1. Example Model 1: Virtual Body Artificial Potential

The cooperative swarm forces $F_{S}$ are defined with the Virtual Body Artificial Potential of Section 2.1 with parameters α, $d_{0}$ and $d_{1}$. In lieu of a potential for the virtual leaders, we assign the HVU tracking function:

(28) $$f_{HVU}=-\frac{K_{1}\left(x_{i}-y_{0}\right)}{∥x_{i}-y_{0}∥}$$

where $y_{0}\in R^{3}$ is the position of the HVU. The dissipative force $f_{v_{i}}=-K_{2}{\dot{x}}_{i}$ is employed to guarantee stability of the swarm system. $K_{1}$ and $K_{2}$ are positive constants. The swarm’s collision avoidance response to the defenders is defined by Equation (4) with parameters $\alpha_{h}$, $h_{0}$ and $h_{1}$. Since there is only a repulsive force between swarm members and defenders, not an attractive force, we set $h_{1}=h_{0}$. For attrition, we use the the damage function defined in Equation (21) of [20]:

(29) $$F_{D}=\lambda \Phi \left(\frac{F-ar^{2}}{\sigma }\right),\;r={∥x_{i}-y_{j}∥}_{2}$$

where Φ is the cumulative normal distribution and ${∥\cdot ∥}_{2}$ is the vector 2-norm. This function smoothly penalizes proximity, with the impact decreasing with distance. The parameters λ, *F*, *a*, and σ shape the steepness of this function and the decline of damage over distance. For the damage rate of defenders inflicted on attackers, we calibrate by the parameters $\lambda_{D}$, $\sigma_{D}$. For the damage rate of attackers inflicted on defenders, we calibrate by the parameters $\lambda_{A}$, $\sigma_{A}$. In both cases, the parameters *F* and *a* in [20] are set to F=0, a=1. Table 1 provides the parameter values that remain fixed in each simulation, and and Table 2 provides the parameters we consider as uncertain.

**Figure 3:**
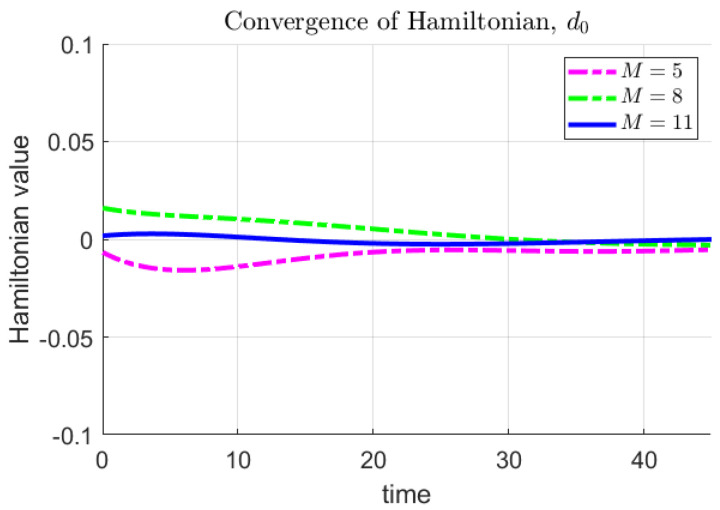
Convergence of Hamiltonion as number of parameter nodes *M* increases.

We first use the nominal parameter values provided in Table 1 and Table 2 to find a nominal solution defender trajectories that result in the minimum probability of HVU destruction. With the results of these simulations as a reference point, we consider as uncertain each of the parameters that define attacker swarm model and weapon capabilities. In this simulation, these parameters are considered individually. The number of discretization nodes for parameter space was chosen by examination of the Hamiltonian. To illustrate this method and the results obtained in Section 4 we compute Hamiltonians for the Problem **SD** and Model 1 with $\theta =d_{0},d_{0}\in \left[0.5,1.5\right]$ and M=[5,8,11]. As *M* increases the sequence of Hamiltonians should converge to the optimal Hamiltonian for the Problem **SD**. For Problem **SD** that should result in a constant, zero-valued Hamiltonian. Figure 3 shows the respective Hamiltonians for M=[5,8,11]. The value M=11 was chosen for simulations, based on the approximately zero-valued Hamiltonian it generates.

**Figure 4:**
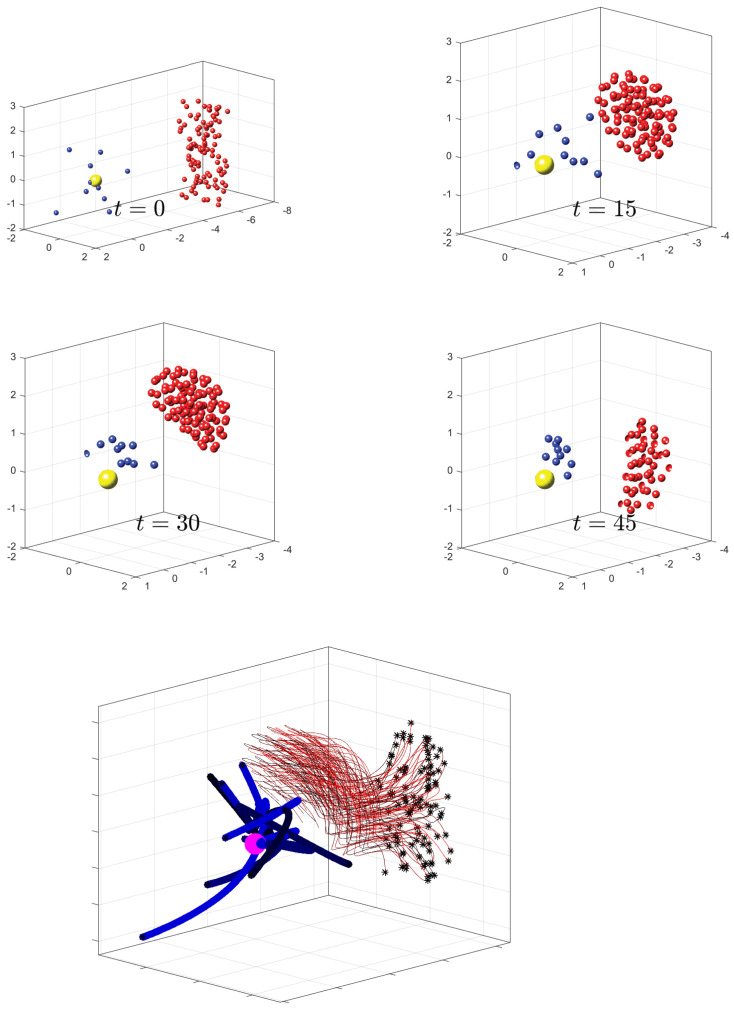
Shown are four snapshots during a simulations at t=0, 15, 30, and 45 (time units are arbitrary). Defenders are represented by blue spheres and attackers by red spheres. The HVU is the yellow sphere. Below these snapshots, we show full trajectories for the entire simulation, which is the result of an optimization protocol using the parameters shown in Table 1.

**Figure 5:**
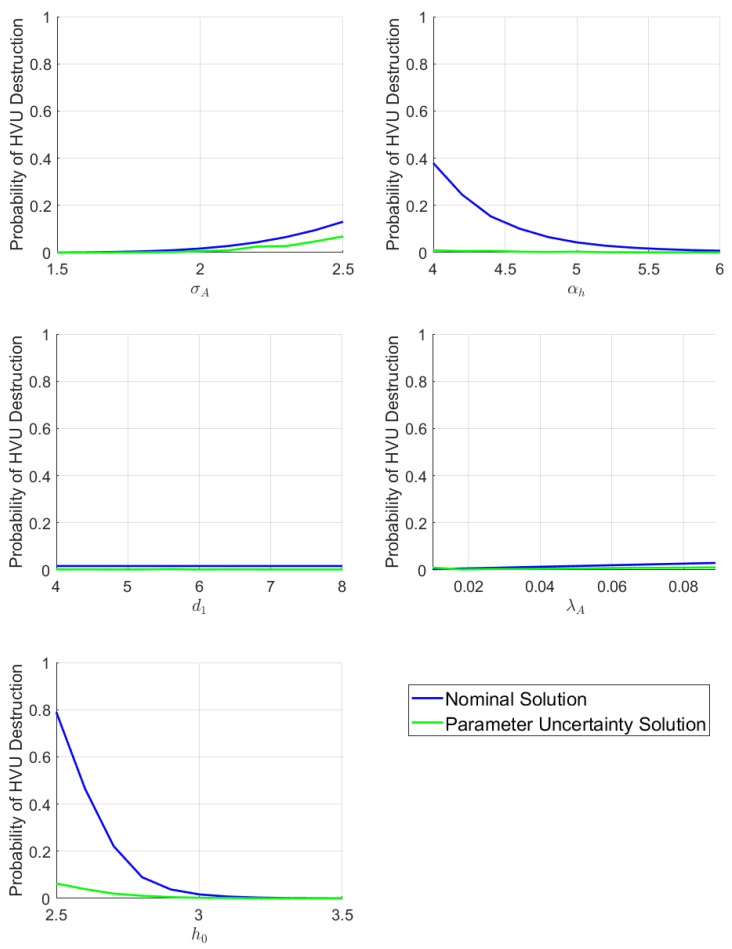
Performance of Solutions of Swarm Model 1 as parameter values are varied. Each panel illustrates a different varied parameter, stated on the *x*-axis.

We compare the performance of the solution generated using uncertain parameter optimal control Problem **SD** versus a solution obtained with the nominal values. Figure 4 shows the nominal solution trajectories. The comparitive results of the nominal solutions vs the uncertain parameter control solutions are shown in Figure 5, where the performance of each is shown for different parameters values.

As seen in Figure 5 the trajectories generated by optimization using the nominal values perform poorly over a range of α, $d_{0}$, $\sigma_{A}$, $\alpha_{k}$ and $h_{0}$. In the case of $h_{0}$, for example, this is because the attackers are less repelled by the defenders when $h_{0}$ is decreased, and they are more able to destroy the HVU from a longer distance as $\sigma_{A}$ is increased. The parameter uncertainty solution, however, demonstrates that using the uncertain parameter optimal control framework a solution can be provided which is robust over a range of parameter values. We contrast these results with the case of uncertain parameters $d_{1}$ and $\lambda_{A}$, also shown in Figure 5. It can be seen that robustness improvements are modest to non-existent for these parameters. This suggests an insensitivity of the problem $d_{1}$ and $\lambda_{A}$ parameters. This kind of analysis can be used to guide inference and observability priorities.

### 5.2. Example Model 2: Reynolds Boid Model

**Figure 6:**
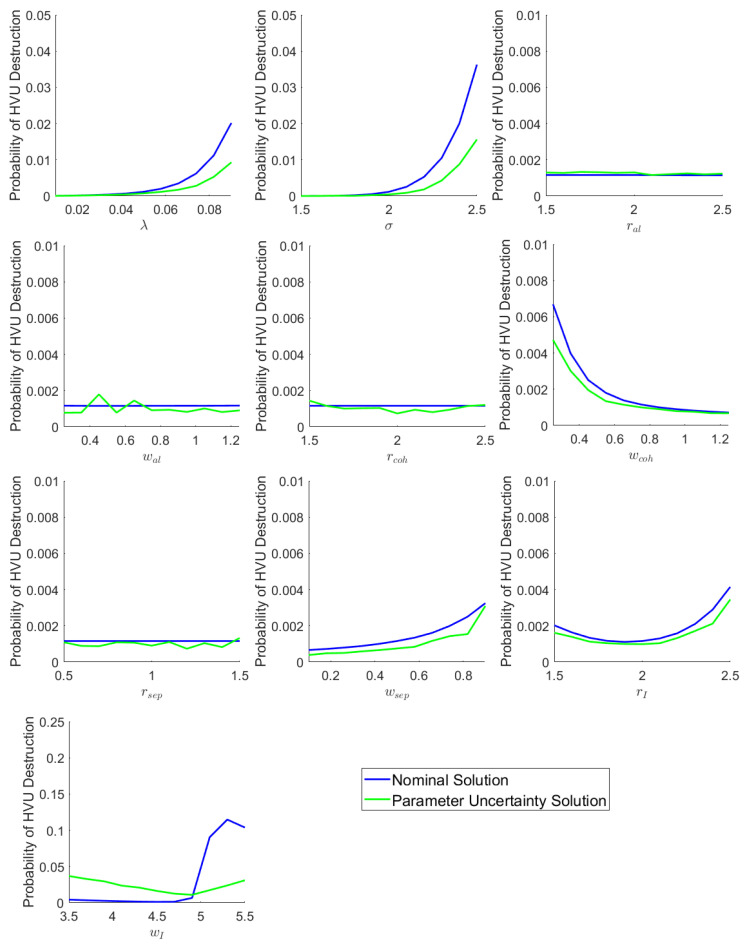
Performance of Solutions of Swarm Model 2 as parameter values are varied.

To demonstrate flexibility of the proposed framework to include diverse swarm models we have applied the same analysis as was done in Section 5.1 to the Reynolds Boid Model introduced in Section 2.1. We apply the same HVU tracking function as Equation (28). The herding force $F_{D}$ of the defenders repelling attackers is applied as a separation force in the form of Equation (10). The fixed parameter values are the same as those in Table 1; the uncertain parameters and ranges are given in Table 3. The results are shown in Figure 6. Again, we see that the tools developed in this paper can be used to gain an insight into the robustness properties of the nominal versus uncertain parameter solutions. For example, we can see that the uncertain parameter solutions perform much better than the nominal ones for the cases where λ, σ and $w_{I}$ are uncertain.

## 6. Conclusions

In this paper, we have built on our previous work on developing an efficient numerical framework for solving uncertain parameter optimal control problems. Unlike uncertainties introduced into systems due to stochastic “noise”, parameter uncertainties do not average or cancel out in regard to their effects. Instead, each possible parameter value creates a specific profile of possibility and risk. The uncertain optimal control framework which has been developed for these problems exploits this inherent structure by producing answers which have been optimized over all parameter profiles. This approach takes into account the possible performance ranges due to uncertainty, while also utilizing what information is known about the uncertain features, such as parameter domains and prior probability distributions over the parameters. Thus, we are able to contain risk, while providing plans which have been optimized for performance under all known conditions. The results reported in this paper include analysis of the consistency of the adjoint variables of the numerical solution. In addition, the paper includes a numerical analysis of a large scale adversarial swarm engagement that clearly demonstrates the benefits of using the proposed framework.

There are many directions for future work for the topics of this paper. The numerical simulations in this paper consider the parameters individually, as one-dimensional parameter spaces. However, Problem **P** allows for multi-dimensional parameter spaces. A more dedicated implementation, taking advantage of the parallelizable form of Equation (16), for example, could certainly manage several simultaneous parameters. Exponential growth as parameter space dimension increases is an issue for both the quadrature format of Equation (15) and handling of the state space size for Equation (16). This can be somewhat mitigated by using sparse grid methods for high-dimensional integration to define the nodes in Equation (15). For large enough sizes, Monte Carlo sampling, rather than quadrature might be more appropriate for designating parameter nodes.

A further direction for future work would be to incorporate these methods into the design of more responsive closed-loop control solutions. The optimization methods in this paper provide open-loop controls. While useful, closed-loop controls would be more ideal for dynamic situations with uncertainty. There are many ways, however, that open-loop solutions can provide stepping stones to developing closed-loop solutions. For instance, Ref. [26] utilizes closed-loop solutions to train a neural network to learn an optimal closed-loop control strategy. Open-loop solutions can also be used to provide initial guesses to discretized closed-loop optimizations, seeding the optimization algorithm.

Another direction for future work is in the greater application of the duality results of Section 4. The numerical results in this paper simply utilize the Hamiltonian consistency. The proof of Theorem 1, however, additionally demonstrates the consistency of the adjoint variables for the problem. As the results demonstrate, parameter sensitivity for these swarm models is highly non-linear. The numerical solutions of Section 5 are able to demonstrate this sensitivity by applying the solution to varied parameter values. However, this is actually a fairly expensive method for a large swarm, as it involves re-evaluation of the swarm ODE for each parameter value. More importantly, it would not be scalable to high-dimensional parameter spaces, as the exponential growth of that approach to sensitivity analysis would be unavoidable. The development of an analytical adjoint sensitivity method for this problem could be of great utility for paring down numerical simulations to only focus on the parameters most relevant to success.

## Figures and Tables

**Table 1:** Model 1 Fixed Parameter Values.

Parameter	Value	Meaning
$t_{f}$	45	final time
$K_{1}$	5	tracking coefficient
*K*	10	number of defenders
$h_{1}$	$h_{0}$	interaction parameter
$\lambda_{D}$	2	defender weapon intensity
$\sigma_{D}$	2	defender weapon range
*N*	100	number of attackers
$K_{2}$	5	dissipative force

**Table 2:** Model 1 Varied Parameter Values.

Parameter	Nominal	Range	Meaning
α	0.5	[0.1, 0.9]	control gain
$d_{0}$	1	[0.5, 1.5]	lower range limit
$d_{1}$	6	[4, 8]	upper range limit
$\lambda_{A}$	0.05	[0.01, 0.09]	weapon intensity
$\sigma_{A}$	2	[1.5. 2.5]	weapon range
$\alpha_{h}$	6	[5, 7]	herding intensity
$h_{0}$	3	[2, 4]	herding range

**Table 3:** Model 2 Varied Parameter Values.

Parameter	Nominal	Range	Meaning
$\lambda_{A}$	0.05	[0.01, 0.09]	weapon intensity
$\sigma_{A}$	2	[1.5, 2.5]	weapon range
$r_{al}$	2	[1.5, 2.5]	alignment range
$w_{al}$	0.75	[0.25, 1.25]	alignment intensity
$r_{coh}$	2	[1.5, 2.5]	cohesion range
$w_{coh}$	0.75	[0.25, 1.25]	cohesion intensity
$r_{sep}$	1	[0.5, 1.5]	separation range
$w_{sep}$	0.5	[0.1, 0.9]	separation intensity
$r_{I}$	2	[1.5, 2.5]	herding range
$w_{I}$	4.5	[3.5, 5.5]	herding intensity

## Data Availability

Not applicable.

## Appendix A. Assumptions and Definitions

We we impose the assumptions in Section 2 of [4]. The definition of accumulation point used in the following proof can be found in Definition 3.2 of [4]. The following assumption is placed on the choice of numerical integration scheme to be utilized in approximating Problem P:

**Assumption** **A1.**
*For each M∈N, there is a set of nodes ${\left\{\theta_{i}^{M}\right\}}_{i=1}^{M}\subset \Theta$ and an associated set of weights ${\left\{\alpha_{i}^{M}\right\}}_{i=1}^{M}\subset R$, such that for any continuous function h:Θ→R,*

$$\begin{array}{c} \int_{\Theta }h\left(\theta \right)d\theta =\lim_{M\to \infty }\sum_{i=1}^{M}h\left(\theta_{i}^{M}\right)\alpha_{i}^{M}. \end{array}$$

This is the same as Assumption 3.1 of [4]; we include it for reference.

In additions to the assumptions of [4], we also impose the following:

**Assumption** **A2.**
*The functions f and r are $C^{1}$. The set *Θ* is compact and $x_{0}:\Theta \mapsto R^{n_{x}}$ is continuous. Moreover, for the compact sets X and U defined in Assumptions 2.3 and 2.4 of [4], and for each t∈[0,T], θ∈Θ, the Jacobians $r_{x}$ and $f_{x}$ are Lipschitz on the set X×U, and the corresponding Lipschitz constants $L_{r}$ and $L_{f}$ are uniformly bounded in θ and t. The function F is $C^{1}$ on X for all θ∈Θ; in addition, F and $F_{x}$ are continuous with respect to θ.*

## Appendix B. Main Theorem Proof

The theorem relies on the following lemma:

**Lemma** **A1.**
*Let $\left\{u_{M}\right\}$ be a sequence of optimal controls for Problem $P^{M}$ with an accumulation point $u^{\infty }$ for the infinite set V⊂N. Let $\left(x^{\infty }\left(t,\theta \right),\lambda^{\infty }\left(t,\theta \right)\right)$ be the solution to the dynamical system:*

(A1)
$$\begin{array}{c} \left\{\begin{array}{c} {\dot{x}}^{\infty }\left(t,\theta \right)=f\left(x^{\infty }\left(t,\theta \right),u^{\infty }\left(t\right),\theta \right)\\ {\dot{\lambda }}^{\infty }\left(t,\theta \right)=-\frac{\partial H(x^{\infty }\left(t,\theta \right),\lambda^{\infty }\left(t,\theta \right),u^{\infty }\left(t\right),t,\theta )}{\partial x} \end{array}\right. \end{array}$$

(A2)
$$\begin{array}{c} \left\{\begin{array}{c} x^{\infty }\left(0,\theta \right)=x_{0}\left(\theta \right)\\ \lambda^{\infty }\left(T,\theta \right)=\frac{\partial F(x^{\infty }\left(T,\theta \right),\theta )}{\partial x} \end{array}\right. \end{array}$$

*where H is defined as per Equation (19), and let $\left\{\left(x_{M}\left(t,\theta \right),\lambda_{M}\left(t,\theta \right)\right)\right\}$ for M∈V be the sequence of solutions to the dynamical systems:*

(A3)
$$\begin{array}{c} \left\{\begin{array}{c} {\dot{x}}_{M}\left(t,\theta \right)=f\left(x_{M}\left(t,\theta \right),u_{M}\left(t\right),\theta \right)\\ {\dot{\lambda }}_{M}\left(t,\theta \right)=-\frac{\partial H(x_{M}\left(t,\theta \right),\lambda_{M}\left(t,\theta \right),u_{M}\left(t\right),t,\theta )}{\partial x} \end{array}\right. \end{array}$$

(A4)
$$\begin{array}{c} \left\{\begin{array}{c} x_{M}\left(0,\theta \right)=x_{0}\left(\theta \right)\\ \lambda_{M}\left(T,\theta \right)=\frac{\partial F(x_{M}\left(T,\theta \right),\theta )}{\partial x} \end{array}\right. \end{array}$$

*Then, the sequence $\left\{\left(x_{M}\left(t,\theta \right),\lambda_{M}\left(t,\theta \right)\right)\right\}$ converges pointwise to $\left(x^{\infty }\left(t,\theta \right),\lambda^{\infty }\left(t,\theta \right)\right)$ and this convergence is uniform in θ.*

**Proof.** The convergence of $\left\{x_{M}\left(t,\theta \right)\right\}$ is given by Lemmas 3.4 and 3.5 of [4]. The convergence of the sequence of solutions $\left\{\lambda_{M}\left(t,\theta \right)\right\}$ is guaranteed by the optimality of $\left\{u_{M}\right\}$. The convergence of $\left\{\lambda_{M}\left(t,\theta \right)\right\}$ then follows the same arguments given the convergence of $\left\{x_{M}\left(t,\theta \right)\right\}$, utilizing the regularity assumptions placed on the derivatives of *F*, *r*, and *f* with respect to *x* to enable the use of Lipschitz conditions on the costate dynamics and transversality conditions. □

**Remark** **A1.**
*Note that $\lambda_{M}\left(t,\theta \right)$ is not a costate of Problem $P^{\lambda M}$, since it is a function of θ. However, when $\theta =\theta_{i}^{M}$, then $\lambda_{M}\left(t,\theta_{i}^{M}\right)={\tilde{\lambda }}_{i}^{M}\left(t\right)$, where ${\tilde{\lambda }}_{i}^{M}$ is the costate of Problem $P^{\lambda M}$ generated by the pair of solutions to Problem $P^{M}$, $\left({\tilde{x}}_{i}^{M},u_{M}^{*}\right)$. In other words, the function $\lambda_{M}\left(t,\theta \right)$ matches the costate values at all collocation nodes. Since these values satisfy the dynamics equations of Problem $P^{\lambda M}$, a further implication of this is that the values of $\lambda_{M}\left(t,\theta_{i}^{M}\right)$ produce feasible solutions to Problem $P^{\lambda M}$.*

**Remark** **A2.**
*Since the functions $\left\{\left(x_{M}\left(t,\theta \right),\lambda_{M}\left(t,\theta \right)\right)\right\}$ obey the respective identities $x_{M}\left(t,\theta_{i}^{M}\right)={\tilde{x}}_{i}^{M}\left(t\right)$ and $\lambda_{M}\left(t,\theta_{i}^{M}\right)={\tilde{\lambda }}_{i}^{M}\left(t\right)$, their convergence to $\left(x^{\infty }\left(t,\theta \right),\lambda^{\infty }\left(t,\theta \right)\right)$ also implies the convergence of the sequence of discretized primals and duals, $\left\{{\tilde{X}}_{M}\right\}$ and $\left\{{\tilde{\Lambda }}_{M}\right\}$, to accumulation points given by the relations*

$$\lim_{M\in V}{\tilde{x}}_{i}^{M}\left(t\right)=x^{\infty }\left(t,\theta_{i}^{M}\right),\;\lim_{M\in V}{\tilde{\lambda }}_{i}^{M}\left(t\right)=\lambda^{\infty }\left(t,\theta_{i}^{M}\right)$$

We now prove Theorem 1. Let $\left\{\left(x_{M}\left(t,\theta \right),\lambda_{M}\left(t,\theta \right)\right)\right\}$ for M∈V be the sequence of solutions defined by Equation (A3) and let $\left(x^{\infty }\left(t,\theta \right),\lambda^{\infty }\left(t,\theta \right)\right)$ be the accumulation functions defined by Equation (A1). Incorporating Remarks A1 and A2, we have:

$$\lim_{M\in V}{\tilde{H}}^{M}\left({\tilde{X}}_{M},{\tilde{\Lambda }}_{M},u_{M},t\right)=\;\;\;\;\;\;\;\;\;$$

$$\lim_{M\in V}\sum_{i=1}^{M}\alpha_{i}^{M}\left[{\tilde{\lambda }}_{i}^{M}\left(t\right)f\left({\tilde{x}}_{i}^{M}\left(t\right),u\left(t\right),\theta_{i}^{M}\right)+r\left({\tilde{x}}_{i}^{M}\left(t\right),u\left(t\right),t,\theta_{i}^{M}\right)\right]$$

$$=\lim_{M\in V}\sum_{i=1}^{M}\alpha_{i}^{M}\left[\lambda_{M}\left(t,\theta_{i}^{M}\right)f\left(x_{M}\left(t,\theta_{i}^{M}\right),u\left(t\right),\theta_{i}^{M}\right)+r\left(x_{M}\left(t,\theta_{i}^{M}\right),u\left(t\right),t,\theta_{i}^{M}\right)\right]$$

Due to the results of Lemma A1, and applying Remark 1 of [4] on the convergence of the quadrature scheme for uniformly convergent sequences of continuous functions, we find that:

$$\lim_{M\in V}{\tilde{H}}^{M}\left({\tilde{X}}_{M},{\tilde{\Lambda }}_{M},u_{M},t\right)=\;\;\;\;\;\;\;\;\;$$

$$\int_{\Theta }\left[\lambda^{\infty }\left(t,\theta \right)f\left(x^{\infty }\left(t,\theta \right),u^{\infty }\left(t\right),\theta \right)+r\left(x^{\infty }\left(t,\theta \right),u^{\infty }\left(t\right),t,\theta \right)\right]d\theta =H\left(x^{\infty },\lambda^{\infty },u^{\infty },t\right)$$

thus proving the theorem.
